# Supplementary material for: Estimating psychopathological networks: Be careful what you wish for
Source: PLoS One. 2017 Jun 23;12(6):e0179891. doi: 10.1371/journal.pone.0179891 (PMC5482475; doi:10.1371/journal.pone.0179891)

Loglinear model (N = 10,000,000)

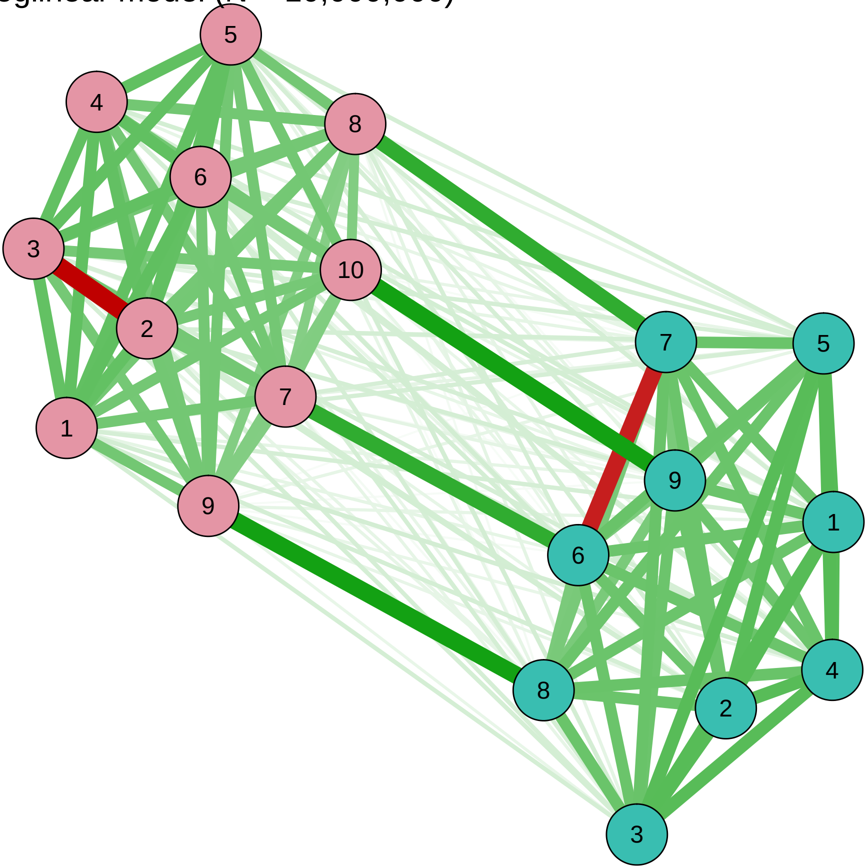

Loglinear model (N = 1,000)

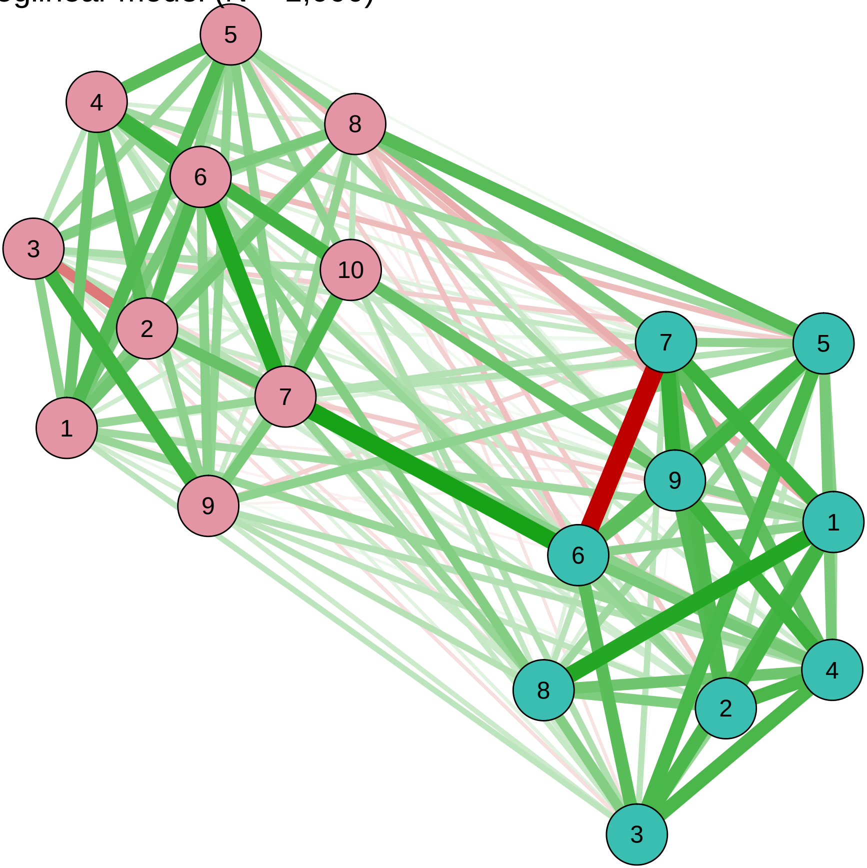

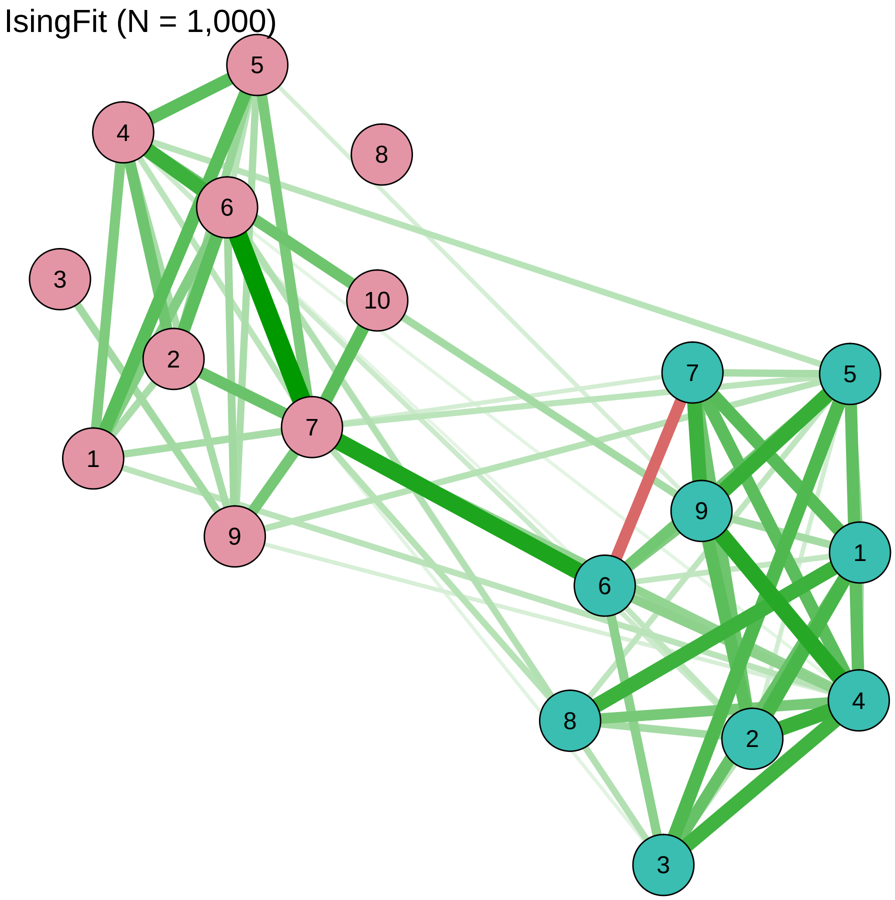
IsingFit (N = 1,000)
5
4
8
6
3
10
2
7
1
9
7
5
9
1
6
8
4
2
3

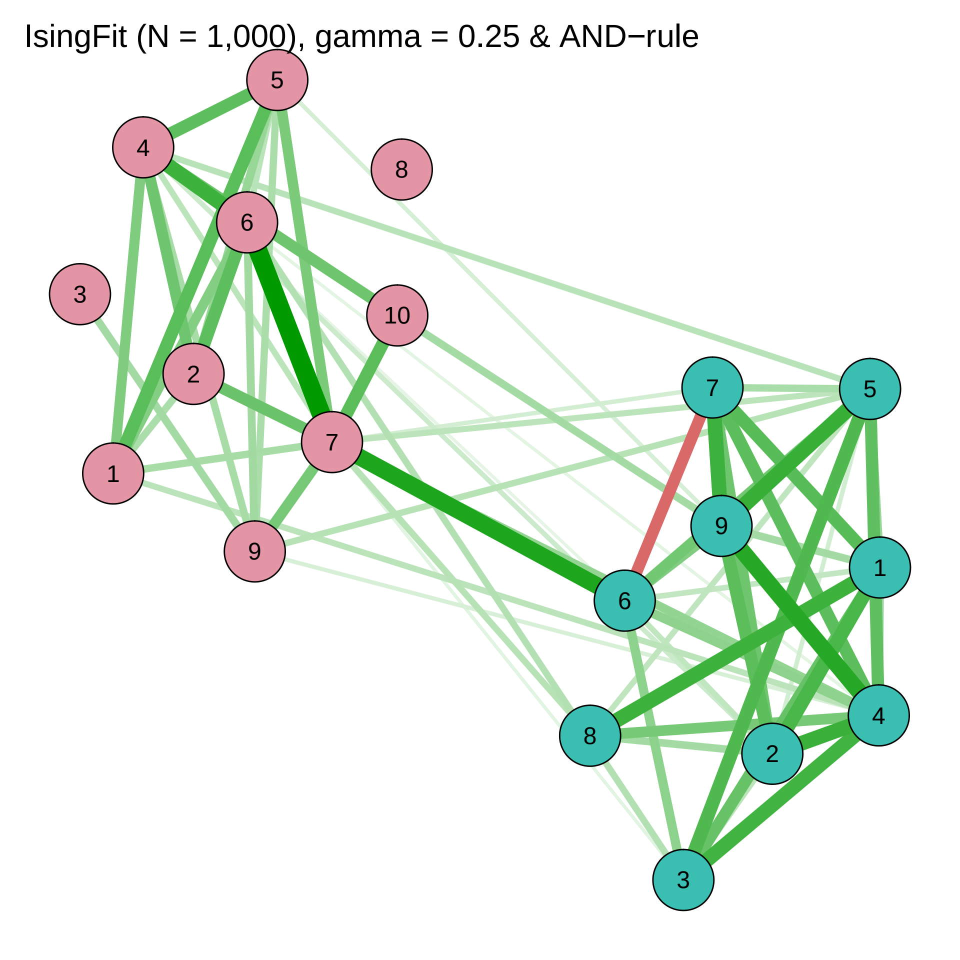
IsingFit (N = 1,000), gamma = 0.25 & AND−rule
5
4
8
6
3
10
2
7
5
1
7
9
1
9
6
4
8
2
3

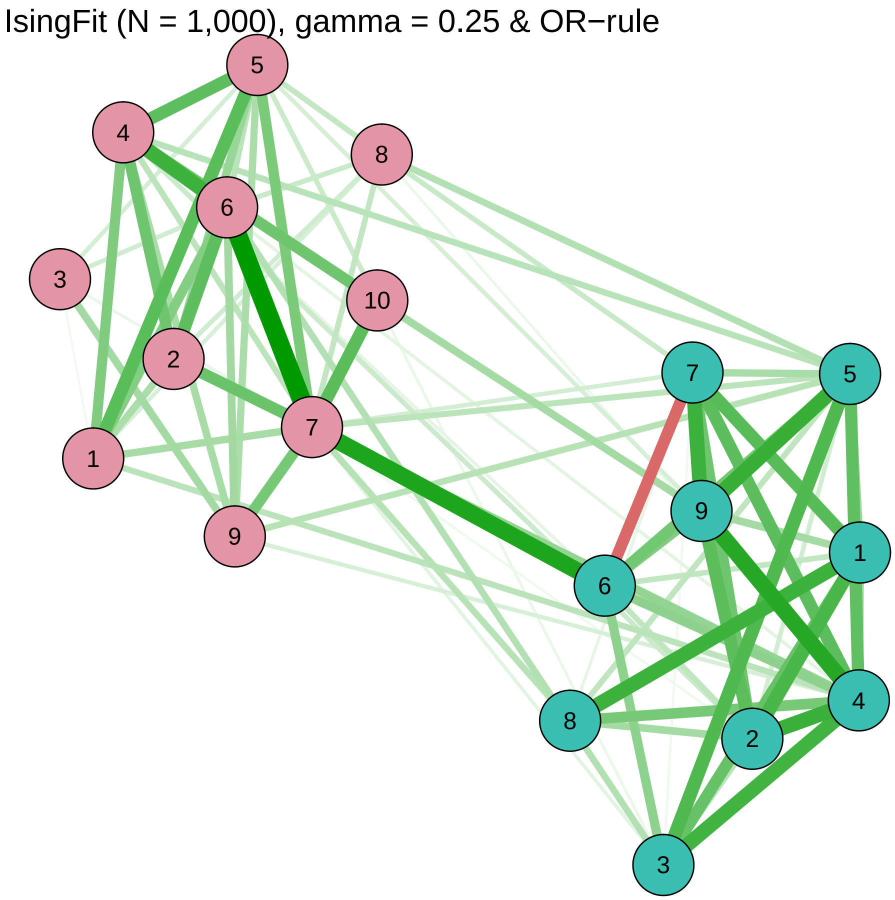
IsingFit (N = 1,000), gamma = 0.25 & OR−rule
5
4
8
6
3
10
2
7
1
9
7
5
9
1
6
4
8
2
3

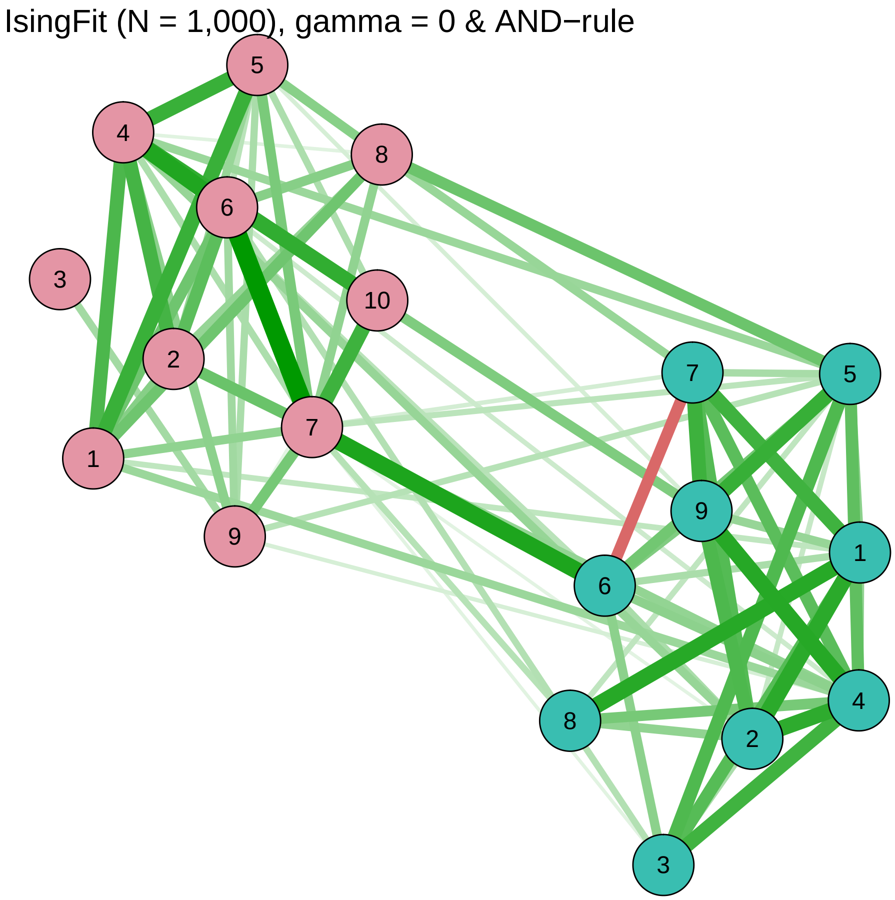
IsingFit (N = 1,000), gamma = 0 & AND−rule
5
4
8
6
3
10
2
7
1
9
7
5
9
1
6
8
4
2
3

Rank 2 approximation (N = 1,000)

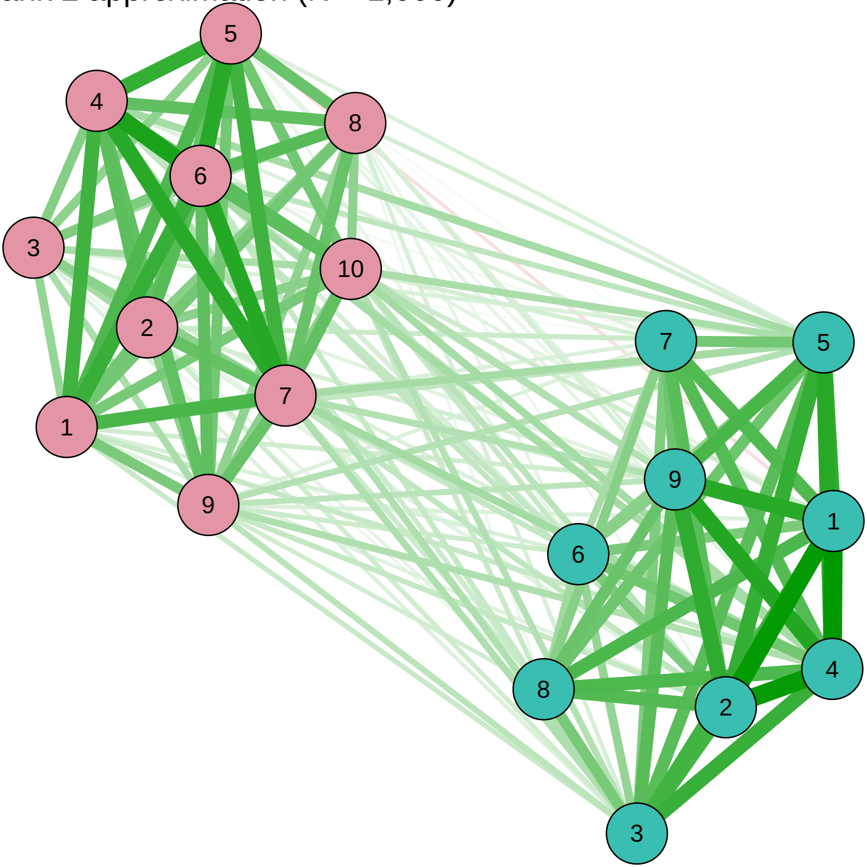

Elastic−net (N = 1,000)

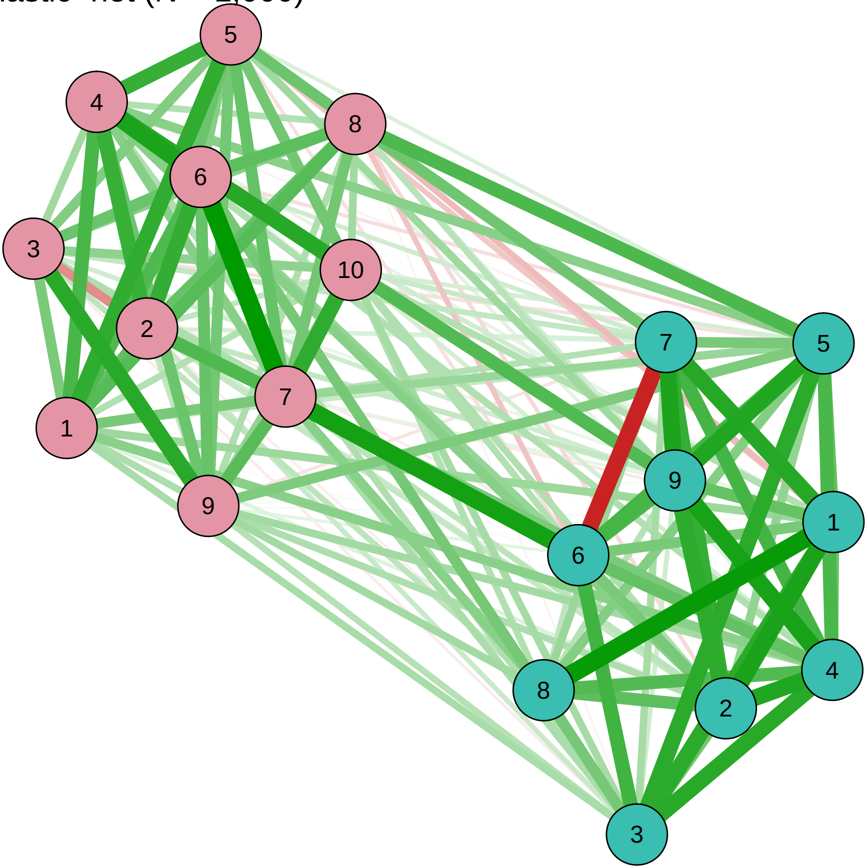

Loglinear model (N = 5,000)

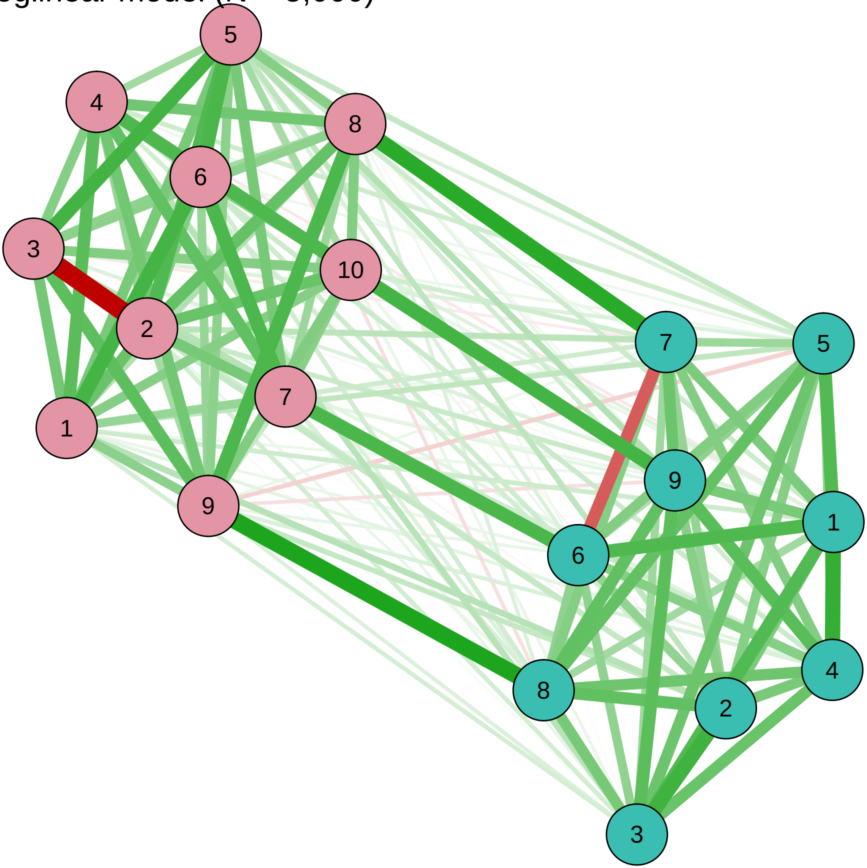

IsingFit (N = 5,000)

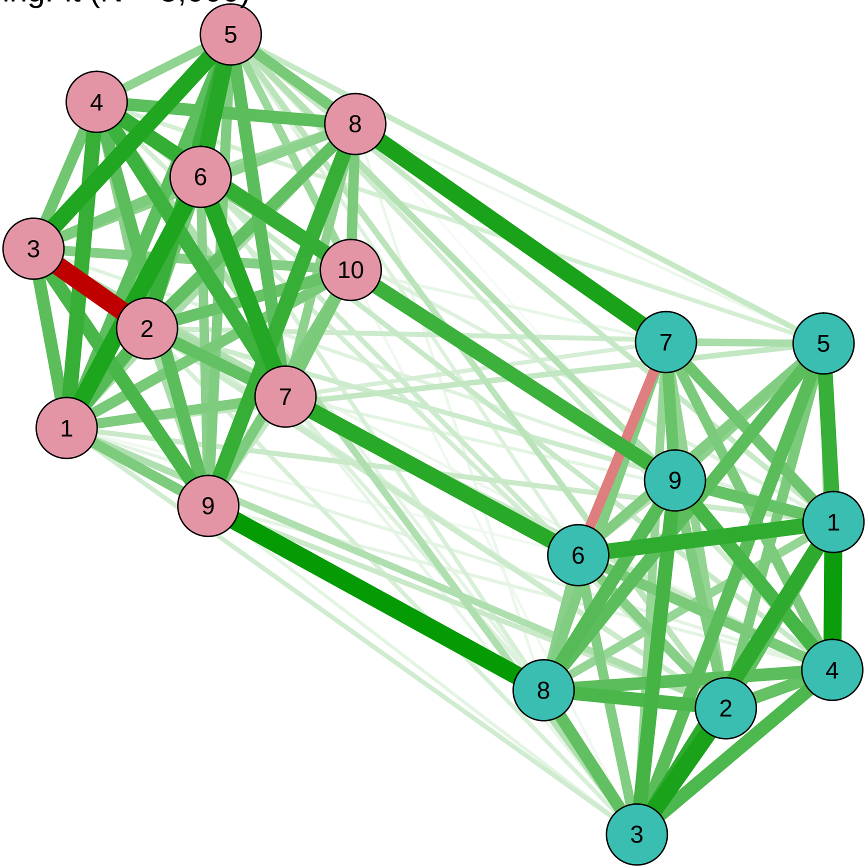

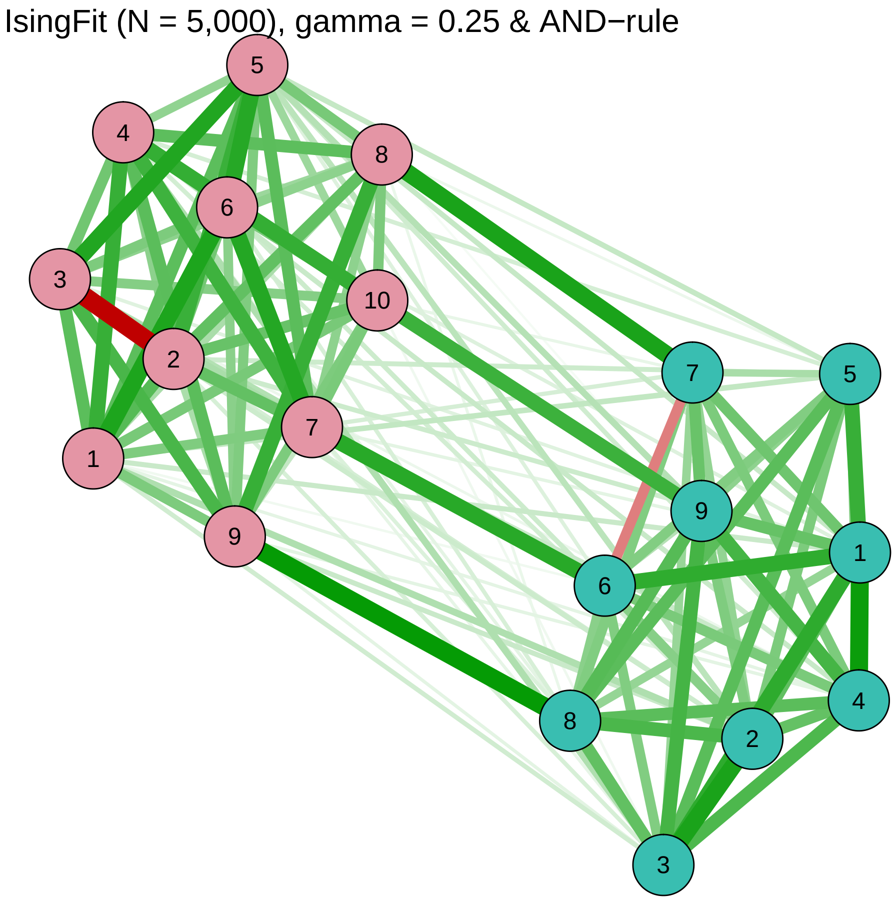
IsingFit (N = 5,000), gamma = 0.25 & AND−rule
5
4
8
6
3
10
2
7
5
7
1
9
9
1
6
4
8
2
3

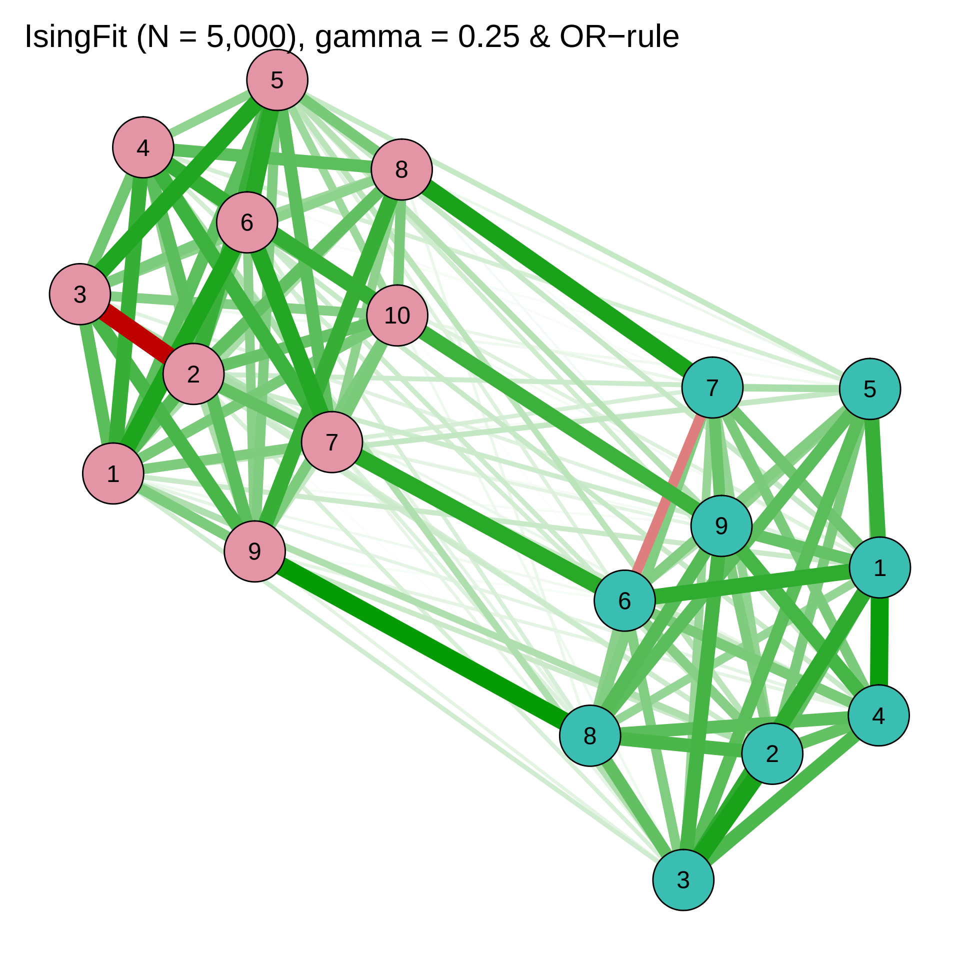
IsingFit (N = 5,000), gamma = 0.25 & OR−rule
5
4
8
6
3
10
2
7
5
7
1
9
9
1
6
8
4
2
3

IsingFit (N = 5,000), gamma = 0 & AND−rule

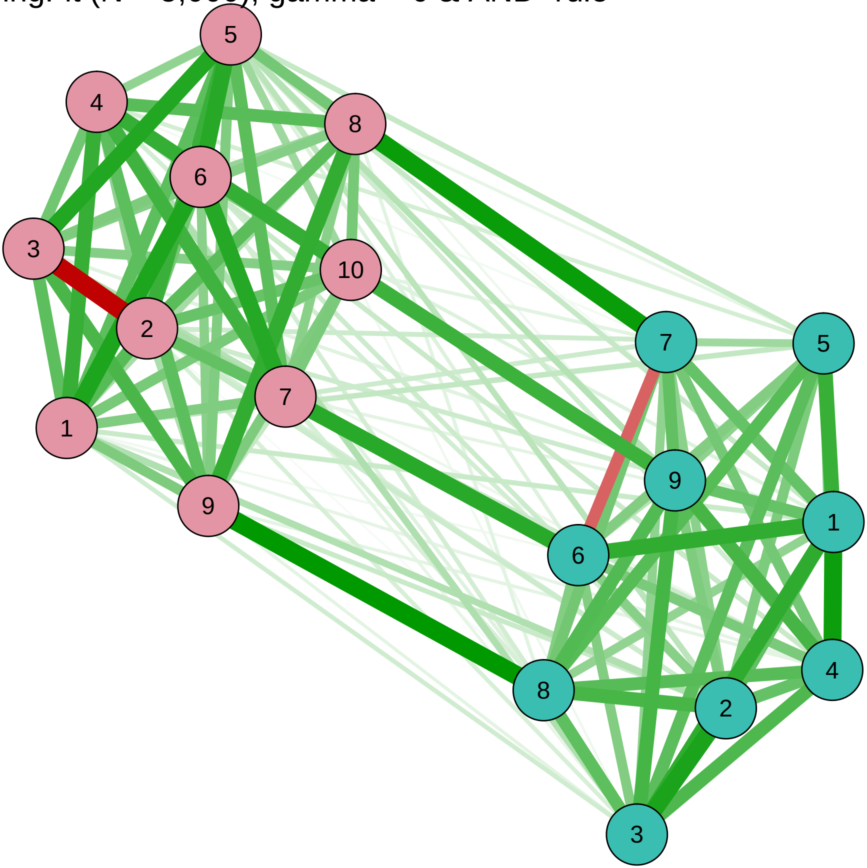

Rank 2 approximation (N = 5,000)

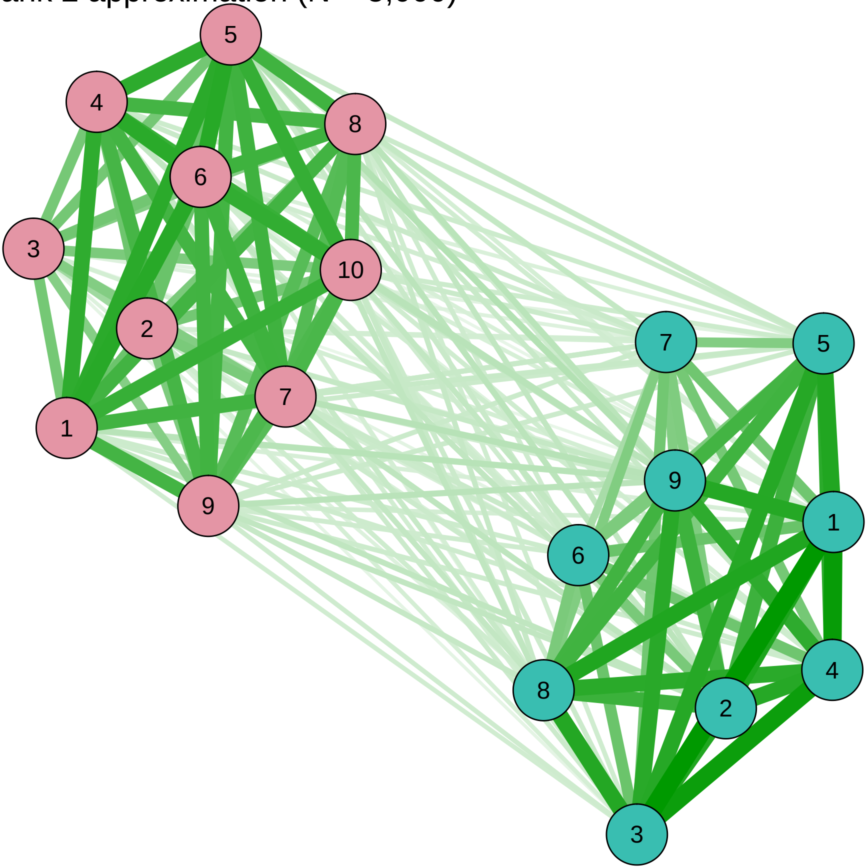

Elastic−net (N = 5,000)

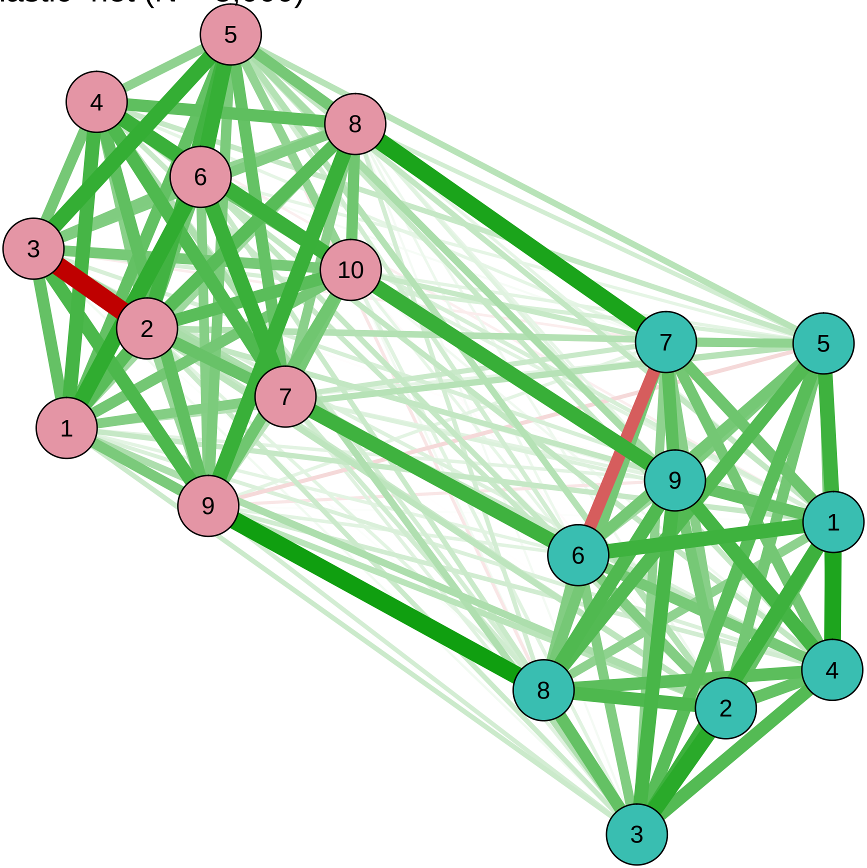

Supplement: S2 File — This PDF file shows more estimated networks based on the model of Fig 4. (PDF) [file pone.0179891.s002.pdf]
